# Molecular ecological network analyses

**DOI:** 10.1186/1471-2105-13-113

**Published:** 2012-05-30

**Authors:** Ye Deng, Yi-Huei Jiang, Yunfeng Yang, Zhili He, Feng Luo, Jizhong Zhou

**Affiliations:** 1 Institute for Environmental Genomics and Department of Botany and Microbiology, University of Oklahoma, Norman, OK 73019, USA; 2 Glomics Inc, Norman, OK 73072, USA; 3 State Key Joint Laboratory of Environment Simulation and Pollution Control, School of Environment, Tsinghua University, Beijing, 100084, China; 4 Earth Sciences Division, Lawrence Berkeley National Laboratory, Berkeley, CA 94720, USA; 5 School of Computing, Clemson University, Clemson, SC 29634, USA

**Keywords:** Ecological network, Random Matrix Theory, Microbial community, Microbiological ecology, Network interaction, Environmental changes

## Abstract

**Background:**

Understanding the interaction among different species within a community and their responses to environmental changes is a central goal in ecology. However, defining the network structure in a microbial community is very challenging due to their extremely high diversity and as-yet uncultivated status. Although recent advance of metagenomic technologies, such as high throughout sequencing and functional gene arrays, provide revolutionary tools for analyzing microbial community structure, it is still difficult to examine network interactions in a microbial community based on high-throughput metagenomics data.

**Results:**

Here, we describe a novel mathematical and bioinformatics framework to construct ecological association networks named molecular ecological networks (MENs) through Random Matrix Theory (RMT)-based methods. Compared to other network construction methods, this approach is remarkable in that the network is automatically defined and robust to noise, thus providing excellent solutions to several common issues associated with high-throughput metagenomics data. We applied it to determine the network structure of microbial communities subjected to long-term experimental warming based on pyrosequencing data of 16 S rRNA genes. We showed that the constructed MENs under both warming and unwarming conditions exhibited topological features of scale free, small world and modularity, which were consistent with previously described molecular ecological networks. Eigengene analysis indicated that the eigengenes represented the module profiles relatively well. In consistency with many other studies, several major environmental traits including temperature and soil pH were found to be important in determining network interactions in the microbial communities examined. To facilitate its application by the scientific community, all these methods and statistical tools have been integrated into a comprehensive Molecular Ecological Network Analysis Pipeline (MENAP), which is open-accessible now (http://ieg2.ou.edu/MENA).

**Conclusions:**

The RMT-based molecular ecological network analysis provides powerful tools to elucidate network interactions in microbial communities and their responses to environmental changes, which are fundamentally important for research in microbial ecology and environmental microbiology.

## Background

In an ecosystem, different species/populations interact with each other to form complicated networks through various types of interactions such as predation, competition and mutualism. On the basis of ecological interactions, ecological networks can be grouped as antagonistic, competitive and mutualistic networks [1]. Traditionally, food webs have been intensively studied in ecological research because they are critical to study the complexity and stability of ecological communities [2,3]. Recent studies showed that food webs possessed typical properties of network topology (e.g. degree distribution, small world effect) [1,4,5]. Within the last decade, mutualistic networks have also been intensively studied [6]. But, it appears that no studies have been performed to examine competitive networks. This is most likely because the network structure is not available based on competitive interactions. Unlike food webs and plant-animal mutualistic networks where the structure is already known, quantifying competitive interactions among different species/populations within a given habitat is difficult so that the network structure for competitive interactions is unknown. This is also true for network studies in microbial ecology. Because of their vast diversity, as-yet uncultivated status [7] and of the lack of appropriate theoretical frameworks and experimental data, very few community-scale network studies have been performed in microbial ecology.

Various network approaches have been developed and widely applied in genomic biology [8]. To reveal the interactions among biological molecules including genes and proteins, differential equation-based network methods [9-12], Bayesian network methods [13,14], and relevance/co-expression network methods [15-20], have been used to infer cellular networks based on gene expression data. Among them, the correlation-based relevance network method is most commonly used largely due to its simple calculation procedure and noise tolerance [21]. However, most studies involving relevance network analysis use arbitrary thresholds, and thus the constructed networks are subjective rather than objective [8]. This problem has been solved by our recent development of a random matrix theory (RMT)-based approach, which is able to automatically identify a threshold for cellular network construction from microarray data [22-24]. Our results showed that the developed novel RMT-based approach can automatically identify cellular networks based on microarray data. Our results also indicated that this approach is a reliable, sensitive and robust tool for identifying transcriptional networks for analyzing high-throughput genomics data for modular network identification and gene function prediction [22,23].

High-throughput technologies such as microarrays and high throughout sequencing have generated massive amounts of data on microbial community diversity and dynamics across various spatial and temporal scales [25,26]. These data offer an unprecedented opportunity to examine interactions among different microbial populations [7]. Recently, a novel conceptual framework, termed molecular ecological networks (MENs), has been proposed and applied to characterize microbial communities in response to elevated CO_2_[27,28]. Here, we provide detailed mathematical and bioinformatic foundation of this novel approach, and further applications to characterize microbial community network interactions in response to long-term experimental warming. Additionally, we provide an online tool, named the Molecular Ecological Network Analyses Pipeline (MENAP), which is freely accessible to the scientific community.

## Results

### Overview of MENA

An ecological network is a representation of various biological interactions (e.g., predation, competition, mutualism) in an ecosystem, in which species (nodes) are connected by pairwise interactions (links) [1,29-32]. As previously described, we refer such molecule-based ecological networks in microbial communities as molecular ecological networks (MENs) [27,28], in which different nodes (molecular markers, e.g., OTUs, functional genes, intergenic regions) are linked by edges (i.e., interactions). The MENs derived from functional gene markers are referred as functional molecular ecological networks (fMENs) [27] and those based on phylogenetic gene markers as phylogenetic molecular ecological networks (pMENs) [28].

**Figure 1:**
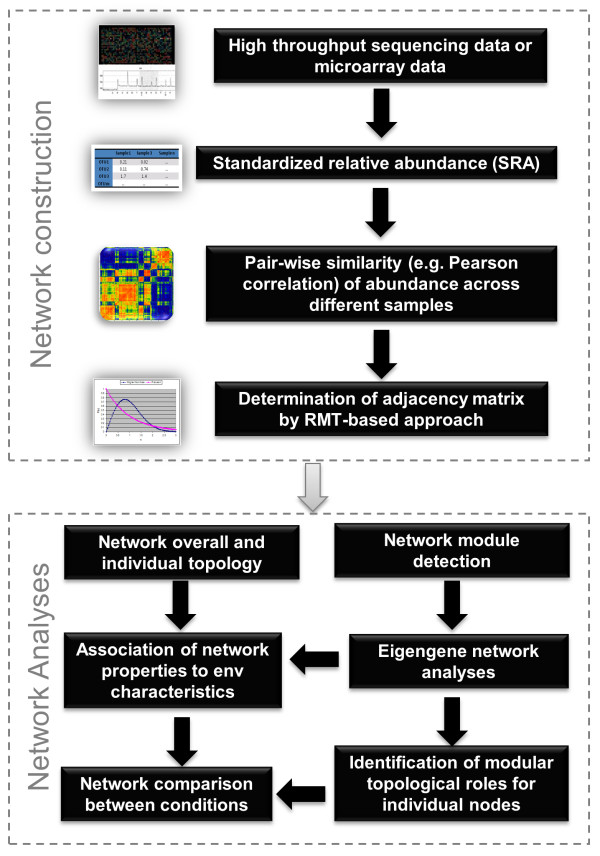
**Overview of the Random Matrix Theory (RMT)-based molecular ecological network analysis**. Two major parts are included, network construction and network analyses. In each of them, several key steps are outlined.

**Figure 2:**
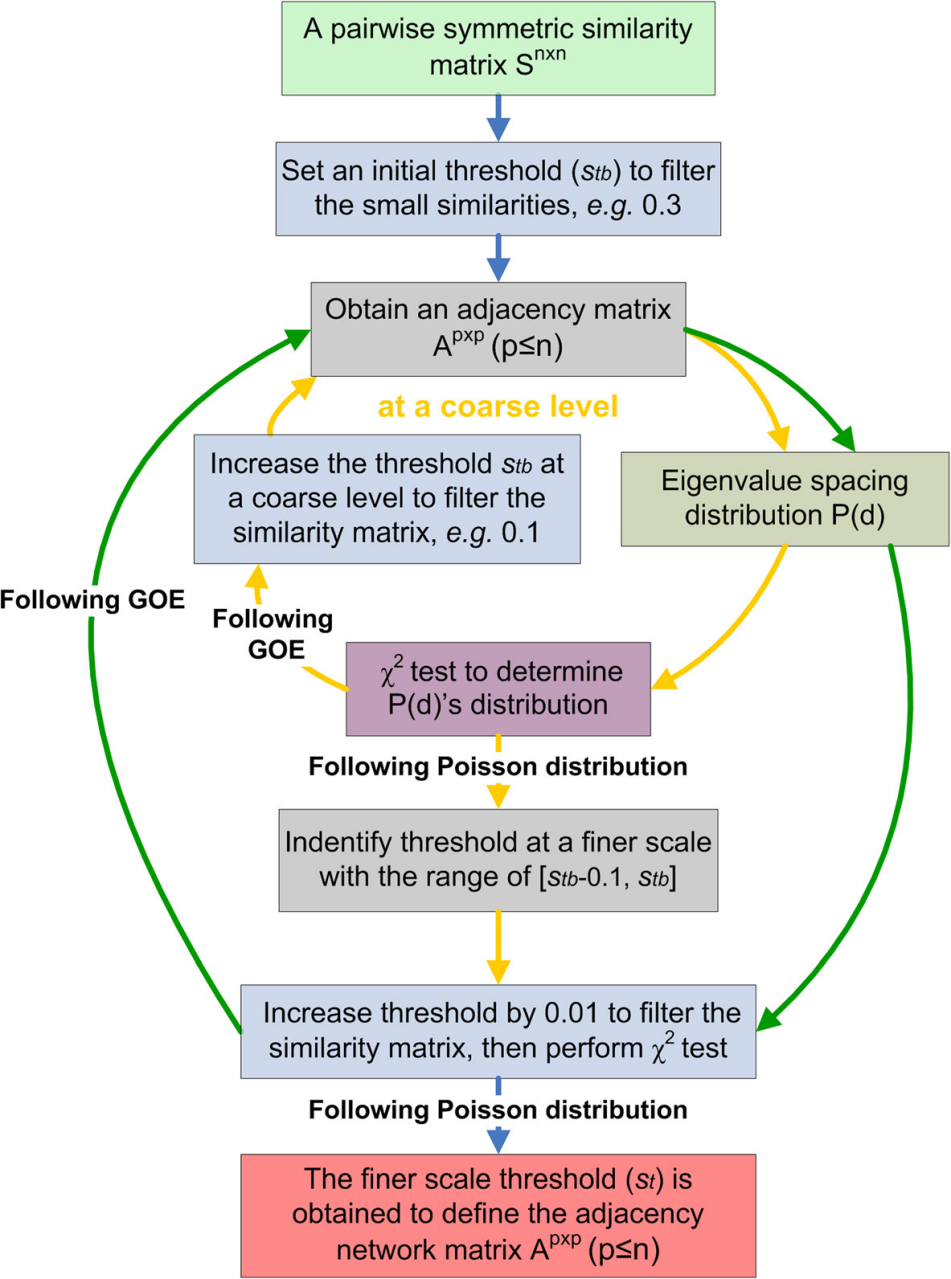
Process of random matrix theory-based approach for automatically detecting threshold to construct molecular ecological networks.

The whole process of MENA can be divided into two phases and each phase is comprised of several major steps (Figure 1). The first phase is network construction, which includes four major steps: data collection, data transformation/standardization, pair-wise similarity matrix calculation, and the adjacent matrix determination by RMT-based approach. Among them, the last step is the key to RMT-based network construction (Figure 2), which has been well established in biological systems [22,23]. Once the adjacency matrix is defined, an undirected network graph can be drawn. The second phase of MENA is network analyses, which is composed of network topology characterization (Table 1,2), module detection, module-based eigengene analysis and identification of modular roles. These methods are important for revealing the networks’ overall and modular organizations and identifying key populations at OTU level. In addition, eigengene network analysis can be performed to reveal higher order organization of MENs, and the associations of network properties to environmental characteristics can be established. Finally, the network differences can be compared under different conditions to analyze how environments affect network structure and interactions.

**Table 1:** The network topological indexes used in this study

**Indexes**	**Formula**	**Explanation**	**Note**	**Ref**
**Part I: network indexes for individual nodes**				
Connectivity	$k_{i}=\sum_{j\neq i}a_{\mathrm{ij}}$	$a_{\mathrm{ij}}$is the connection strength between nodes i and j.	It is also called node degree. It is the most commonly used concept for desibing the topological property of a node in a network.	[33]
Stress centrality	$SC_{i}=\sum_{jk}\sigma (j,i,k)$	σ(j,i,k) is the number of shortest paths between nodes *j* and *k* that pass through node *i*.	It is used to desibe the number of geodesic paths that pass through the i^th^ node. High Stress node can serve as a broker.	[34]
Betweenness	$B_{i}=\sum_{jk}\frac{\sigma (j,i,k)}{\sigma (j,k)}$	σ(j,k) is the total number of shortest paths between *j* and *k*.	It is used to desibe the ratio of paths that pass through the i^th^ node. High Betweenness node can serve as a broker similar to stress centrality.	[34]
Eigenvector centrality	$EC_{i}=\frac{1}{\lambda }\sum_{j\in M(i)}EC_{j}$	*M(i)* is the set of nodes that are connected to the i^th^ node and *λ* is a constant eigenvalue.	It is used to desibe the degree of a central node that it is connected to other central nodes.	[35]
Clustering coefficient	CCi=2liki'(ki'−1)	*l*_*i*_ is the number of links between neighbors of node *i* and *k*_*i*_*’* is the number of neighbors of node *i.*	It desibes how well a node is connected with its neighbors. If it is fully connected to its neighbors, the clustering coefficient is 1. A value close to 0 means that there are hardly any connections with its neighbors. It was used to desibe hierarchical properties of networks.	[36,37]
Vulnerability	$V_{i}=\frac{E-E_{i}}{E}$	*E* is the global efficiency and *E*_*i*_ is the global efficiency after the removal of the node *i* and its entire links.	It measures the deease of node i on the system performance if node i and all associated links are removed.	[38]
**Part II: The overall network topological indexes**				
Average connectivity	$avgK=\frac{\sum_{i=1}^{n}k_{i}}{n}$	*k*_*i*_ is degree of node *i* and *n* is the number of nodes.	Higher *avgK* means a more complex network.	[39]
Average geodesic distance	$GD=\frac{1}{n(n-1)}\sum_{i\neq j}d_{\mathrm{ij}}$	*d*_*ij*_ is the shortest path between node *i* and *j*.	A smaller *GD* means all the nodes in the network are closer.	[39]
Geodesic efficiency	$E=\frac{1}{n(n-1)}\sum_{i\neq j}\frac{1}{d_{\mathrm{ij}}}$	all parameters shown above.	It is the opposite of *GD*. A higher *E* means that the nodes are closer.	[40]
Harmonic geodesic distance	$HD=\frac{1}{E}$	*E* is geodesic efficiency.	The reciprocal of *E*, which is similar to *GD* but more appropriate for disjoint graph.	[40]
Centralization of degree	$CD=\sum_{i=1}^{n}\left(\max(k)-k_{i}\right)$	max(*k*) is the maximal value of all connectivity values and *k*_*i*_ represents the connectivity of *i*^th^ node. Finally this value is normalized by the theoretical maximum centralization score.	It is close to 1 for a network with star topology and in contrast close to 0 for a network where each node has the same connectivity.	[41]
Centralization of betweenness	$CB=\sum_{i=1}^{n}\left(\max(B)-B_{i}\right)$	max(*B*) is the maximal value of all betweenness values and *B*_*i*_ represents the betweenness of *i*^th^ node. Finally this value is normalized by the theoretical maximum centralization score.	It is close to 0 for a network where each node has the same betweenness, and the bigger the more difference among all betweenness values.	[41]
Centralization of stress centrality	$CS=\sum_{i=1}^{n}\left(\max(SC)-SC_{i}\right)$	max(*SC*) is the maximal value of all stress centrality values and *SC*_*i*_ represents the stress centrality of *i*^th^ node. Finally this value is normalized by the theoretical maximum centralization score.	It is close to 0 for a network where each node has the same stress centrality, and the bigger the more difference among all stress centrality values.	[41]
Centralization of eigenvector centrality	$CE=\sum_{i=1}^{n}\left(\max(EC)-EC_{i}\right)$	max(*EC*) is the maximal value of all eigenvector centrality values and *EC*_*i*_ represents the eigenvector centrality of *i*^th^ node. Finally this value is normalized by the theoretical maximum centralization score.	It is close to 0 for a network where each node has the same eigenvector centrality, and the bigger the more difference among all eigenvector centrality values.	[41]
Density	$D=\frac{l}{l_{\exp}}=\frac{2l}{n(n-1)}$	*l* is the sum of total links and *l*_*exp*_ is the number of possible links.	It is closely related to the average connectivity.	[41]
Average clustering coefficient	$avgCC=\frac{\sum_{i=1}^{n}CC_{i}}{n}$	$CC_{i}$ is the clustering coefficient of node *i*.	It is used to measure the extent of module structure present in a network.	[36]
Transitivity	Trans=∑i=1n(2li)∑i=1nki'(ki'−1)	*l*_*i*_ is the number of links between neighbors of node *i* and *k*_*i*_*’* is the number of neighbors of node *i.*	Sometimes it is also called the entire clustering coefficient. It has been shown to be a key structural property in social networks.	[41]
Connectedness	$Con=1-\left[\frac{W}{n(n-1)/2}\right]$	*W* is the number of pairs of nodes that are not reachable.	It is one of the most important measurements for summarizing hierarchical structures. *Con* is 0 for graph without edges and is 1 for a connected graph.	[42]

**Table 2:** Common characters of complex networks

**Terminology**	**Explanation**
**Scale-free**	It is a most notable characteristic in complex systems. It was used to desibe the finding that most nodes in a network have few neighbors while few nodes have large amount of neighbors. In most cases, the connectivity distribution asymptotically follows a power law [43]. It can be expressed in $P(k)\sim k^{-y}$, where *P(k)* is the number of nodes with *k* degrees, *k* is connectivity/degrees and *γ* is a constant.
**Small-world**	It is a terminology in network analyses to depict the average distance between nodes in a network is short, usually logarithmically with the total number of nodes [44]. It means the network nodes are always closely related with each other.
**Modularity**	It was used to demonstrate a network which could be naturally divided into communities or modules [45]. Each module in gene regulation networks is considered as a functional unit which consisted of several elementary genes and performed an identifiable task [23,46]. A modularity value can be calculated by Newman’s method [45] which was used to measure how well a network is able to be separated into modules. The value is between 0 to 1.
**Hierarchy**	It was used to depict the networks which could be arranged into a hierarchy of groups representing in a tree structure. Several studies demonstrated that metabolic networks are usually accompanied by a hierarchical modularity [37,44]. It was potentially consistent with the notion that the accumulation of many local changes affects the small highly integrated modules more than the larger, less integrated modules [37]. One of the most important signatures for hierarchical modular organizations is that the scaling of clustering coefficient follows *C*(*k*) ~ *k*^−*γ*^ (scaling law), in which *k* is connectivity and *γ* is a constant [47].

### Molecular network under experimental warming

Here we used 16 S rRNA gene-based pyrosequencing data from a long-term experimental warming site [48] to construct pMENs and demonstrate the whole process of MENA. The experimental site was established in grassland with two atmospheric temperature treatments, ambient (unwarming) and +2 °C warming. Six replicate plots were set up for each treatment. The environmental DNA of microbial community was extracted from the soil samples of those 12 plots and 2 or 3 unique tags with 16 S rRNA gene conserved primers were used to amplify the V4-V5 hypervariable regions of the 16 S rRNA genes. Altogether, there were 14 replicate datasets for each treatment of warming or unwarming. After preprocessing all raw sequences, the numbers of sequences for all 28 samples ranged from 1,033 to 5,498. After defining OTUs within 0.03 sequence difference, an OTU distribution table with 1,417 distinct OTUs across 28 samples was obtained. Since the numbers of sequences of all samples were diverse, the abundance data were transformed into relative abundance by dividing the sum of each sample as described previously [48]. The relative abundance table was split into two datasets: warming and unwarming. For each of the datasets, only the OTUs appeared in 7 or more replicates were used for correlation calculations, resulting in 228 and 197 OTUs for warming and unwarming datasets, respectively. After threshold scanning through RMT-based approach, the phylogenetic molecular ecological networks (pMENs) under warming and unwarming conditions were constructed with an identical similarity threshold 0.76 (Table 3). The final warming and unwarming pMENs included 177 and 152 nodes which had at least one edge, and 279 and 263 total edges, respectively.

**Table 3:** **Topological properties of the empirical molecular ecological networks (MENs) of additional miobial communities and their associated random MENs**^**a**^

Habitats of communities^b^	Empirical networks							Random networks		
	Similarity threshold (*s*_*t*_)	Network size (*n*)	R^2^ of power law	R^2^ of scaling law	Average path (GD)	Average Clustering coefficient (*avgCC*)	Modularity & (the number of modules)	Average path (GD)	Average clustering coefficient (*avgCC*)	Modularity (*M*)
**Functional MENs**										
Grassland soils under elevated CO_2_, MN ^(i)^	0.80	254	0.79	0.25	3.09	0.22	0.44 (18)	3.00 ± 0.03	0.099 ± 0.009	0.31 ± 0.01
Grassland soils under ambient CO_2_, MN ^(i)^	0.80	184	0.88	0.11	4.21	0.10	0.65 (16)	3.84 ± 0.06	0.028 ± 0.007	0.52 ± 0.01
Lake sediment, Lake DePue, WI ^(ii)^	0.92	151	0.85	0.73	3.47	0.09	0.48 (8)	3.46 ± 0.05	0.046 ± 0.010	0.45 ± 0.01
Groundwater, Well 101–2, Oak Ridge, TN ^(iii)^	0.95	107	0.74	0.44	3.12	0.29	0.52 (11)	3.13 ± 0.07	0.081 ± 0.017	0.40 ± 0.01
Groundwater Well 102–2, Oak Ridge, TN ^(iii)^	0.89	140	0.79	0.21	4.22	0.17	0.67 (12)	3.89 ± 0.08	0.033 ± 0.012	0.53 ± 0.01
Groundwater Well 102–3, Oak Ridge, TN ^(iii)^	0.87	117	0.85	0.19	3.57	0.25	0.64 (13)	3.54 ± 0.09	0.049 ± 0.013	0.48 ± 0.01
**Phylogenetic MENs (454 pyrosequencing)**										
Grassland soils under warming, Norman, OK ^(iv)^	0.76	177	0.83	0.48	3.91	0.13	0.67 (18)	3.94 ± 0.20	0.020 ± 0.008	0.44 ± 0.01
Grassland soils under unwarming, Norman, OK ^(iv)^	0.76	152	0.88	0.10	2.71	0.09	0.61 (20)	3.39 ± 0.23	0.038 ± 0.010	0.47 ± 0.01
Grassland soils under elevated CO_2_, MN ^(i)^	0.78	263	0.89	0.26	3.95	0.25	0.81 (34)	3.98 ± 0.22	0.015 ± 0.006	0.61 ± 0.02
Grassland soils under ambient CO_2_, MN ^(i)^	077	292	0.87	0.22	4.26	0.27	0.85 (36)	4.10 ± 0.20	0.017 ± 0.005	0.59 ± 0.01
Agricultural soil, Africa ^(v)^	0.77	384	0.86	0.20	4.99	0.34	0.86 (32)	3.99 ± 0.04	0.020 ± 0.004	0.48 ± 0.01
Human intestine, Stanford, CA ^(vi)^	0.86	215	0.92	0.18	3.55	0.13	0.69 (27)	4.23 ± 0.10	0.025 ± 0.009	0.58 ± 0.01

^a^Various parameters of the empirical networks and generation of random networks are explained in the Table 1.

^b^Sample sources: (i) the grassland soils under elevated and ambient CO_2_ were collected from a free-air CO_2_ enrichment field in Minnesota which were analyzed with both GeoChip3.0 and 16 S pyrosequencing [49]. The fMENs analysis was desibed in Zhou et al. [27] and pMENs analysis was desibed in Zhou et al. [28]. (ii) The lake sediment samples from Lake DePue were analyzed with GeoChip 2.0. (iii) The groundwater samples from three different Wells in Oak Ridge, Tennessee were analyzed with GeoChip 2.0 [50]. (iv) The grassland samples under warming and unwarming were collected from the long term warming experiment at Oklahoma [51] and analyzed with 16 S pyrosequencing [48]. (v) The pyrosequencing data of agricultural soils from Africa and the groundwater samples from Oak Ridge was provided by Dr. Tiedje and his colleagues at Michigan State University. (vi) The human intestine sample from Stanford was desibed elsewhere [52].

### The robustness of MENs to noise

**Figure 3:**
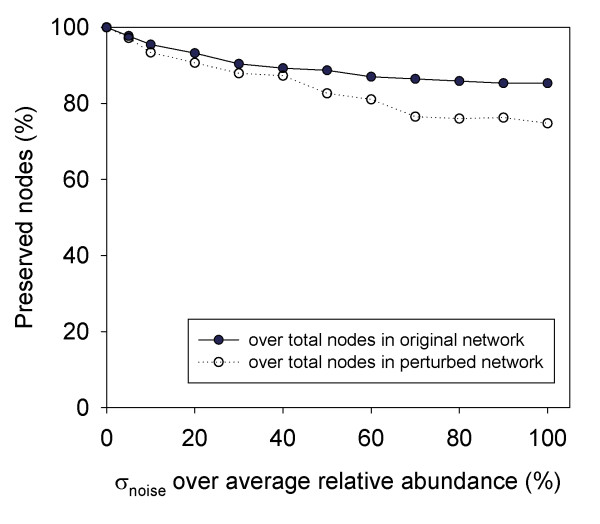
**The robustness to noise of RMT-based MEN construction.** Ineasing levels of Gaussian noise were added to the pyrosequencing datasets under experimental warming. The mean of noise was zero and standard deviation (σ_noise_) was set to 5, 10, 20, 30 to 100 % of the average of relative abundance of whole dataset. The thresholds (*S*_*t*_) of all permutated datasets were set to 0.76 that was consistent with original dataset.

In order to examine the robustness of MEN approach to noise, different levels (1 to 100 % of original standard deviation) of Gaussian noise were added to the warming dataset. Once various levels of noise were added, new correlation matrices based on these noise-added datasets were calculated. The same similarity threshold used for the original datasets was used for defining adjacency matrices in the new datasets. When less than 40 % noise was added, roughly 90 % of the original OTUs were still detected in the perturbed networks (Figure 3). With 100 % Gaussian noise, more than 85 % nodes from original network were still preserved and accounted for 75 % nodes of perturbed network. These results indicate that the RMT-based MEN construction approach is robust to noise.

### The overall MENs topology

Scale-free, small-world, modularity and hierarchy are common network properties in many complex systems (Table 2) [8,53,54]. The overall topology indices (Table 3) revealed that all curves of network connectivity distribution were fitted well with the power-law model (R^2^ values from 0.74 to 0.92), indicative of scale-free networks. Also, the average path lengths (*GD*) were 3.09 to 5.08, which were close to logarithms of the total number of network nodes and comparable to those in other networks displaying small-world behavior, suggesting that the MENs in these microbial communities had the typical property of small world. For modularity, all modularity values (*M*) were from 0.44 to 0.86, which were significantly higher than the *M* values from their corresponding randomized networks, Therefore, all constructed MENs appeared to be modular. Finally, the hierarchy property was examined by the scaling of clustering coefficient. R^2^ values of the linear relationship between logarithms of clustering coefficients and the logarithms of connectivity ranged from 0.10 to 0.73, indicating the hierarchical behavior was quite variable. MENs from certain habitats may have highly hierarchical structures like sediment samples from Lake DePue (0.73), but others may not (Table 3). Overall, our constructed MENs from different habitats clearly exhibit scale free, small world and modularity properties, but hierarchy property is displayed on certain networks.

### Modular structure

Modularity is a very important concept in ecology. It could originate from specificity of interactions (e.g. predation, pollination), habitat heterogeneity, resource partition, ecological niche overlap, natural selection, convergent evolution, and phylogenetic relatedness, and it could be important for system stability and resilience [55]. In MENs, a module in the network is a group of OTUs that are highly connected among themselves, but had much fewer connections with OTUs outside the group. Random matrix theory-based approach is able to delineate separate modules, but some modules could still be very big.

**Figure 4:**
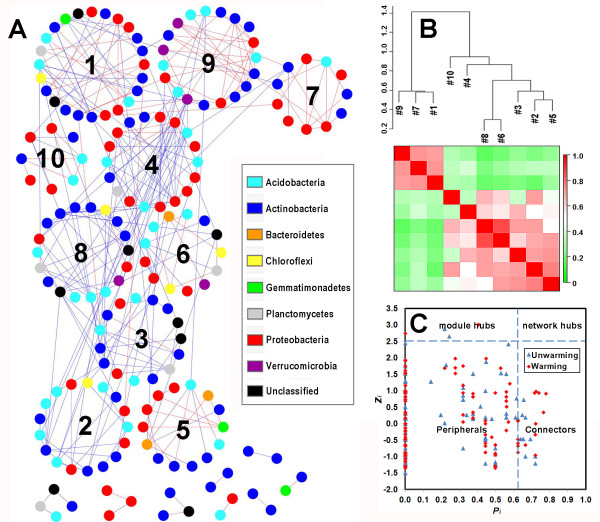
**The submodules of the warming pMEN.** (**A**) The network graph with submodule structure by the fast greedy modularity optimization method. Each node signifies an OTU, which could correspond to a miobial population. Colors of the nodes indicate different major phyla. A blue edge indicates a positive interaction between two individual nodes, while a red edge indicates a negative interaction. (**B**) The correlations and heatmap to show module eigengenes of warming pMEN. The upper part is the hierarchical clustering based on the Pearson correlations among module eigengenes and the below heatmap shows the coefficient values (*r*). Red color means higher correlation whereas green color signified lower correlation. (**C**) ZP-plot showing distribution of OTUs based on their module-based topological roles. Each dot represents an OTU in the dataset of warming (red), or unwarming (green). The topological role of each OTU was determined according to the scatter plot of within-module connectivity (*z*) and among-module connectivity (*P*) [55,60].

We used several methods, including short random walks [56], leading eigenvector of the community matrix [57], simulated annealing approach [58,59] and the greedy modularity optimization [57], to define modules and submodules within a large module. From the evaluation of warming and unwarming pMENs, short random walks generated 27 and 31 modules with *M* values 0.61 and 0.56, respectively; the leading eigenvector of the matrix generated 22 and 28 modules with *M* values 0.61 and 0.54, respectively; the greedy modularity optimization had 18 and 20 modules with *M* values 0.67 and 0.61, respectively; the simulated annealing approach had average 18 and 19 modules with average *M* values 0.67 and 0.61, respectively. From these results, the greedy modularity optimization and simulated annealing approach had higher *M* values than two other approaches, indicating they are more effective in separating the complex networks into submodules. Notably, since the simulated annealing approach was stochastic [59], the submodules of pMENs generated by this approach were slightly different with different runs. Therefore, the greedy modularity optimization approach was preferred to identify the submodular structure of MENs. The modular pMEN of warming pyrosequencing dataset was shown in Figure 4A. A total of 10 joint submodules with ≥8 nodes were isolated from a single large module and all the other isolated modules were relatively small (2 to 4 nodes). The size of modules or submodules varied with 2 to 24 nodes.

### Eigengene network analysis and the modular topological roles

After modules and submodules are determined, the eigengene analysis is used to reveal higher order organizations in the network structure [60-62]. In the eigengene analysis, each module is represented by its singular value decomposition (SVD) of abundance profile called module eigengene [62]. In the warming pMEN, the module eigengenes from top 10 large submodules (≥8 nodes) explained 30 - 68 % variations of relative abundance across different replicates, suggesting that these eigengenes represented the module profiles relatively well. The correlations among module eigengenes were used to define the eigengene network. Eigengene analysis is important for revealing higher order organization and identifying key populations based on network topology [62]. In warming pMEN, these correlations of 10 largest submodules were visualized as a heat-map and hierarchical clustering diagram (Figure 4B). The eigengenes within several groups of submodules showed significant correlations and clustered together as super-groups, such as #6 and #8, #2, #5 and #3, and #1, and #7 and #9, which were referred as meta-modules that exhibit a high order organization among submodules. Besides, within each module, eigengene analysis approach was able to show the representative abundance profile and identify key members as shown in our previous paper [28].

Different nodes play distinct topological roles in the network [33]. The analysis of modular topological roles is important to identify key populations or functional genes based on the nodes’ roles in their own modules. Their topological roles can be defined by two parameters, within-module connectivity (*z*_*i*_) and among-module connectivity (*P*_*i*_). The topological roles of nodes in warming and unwarming pMENs were illustrated in ZP-plot (Figure 4C). According to values of *z*_*i*_ and *P*_*i*_, the roles of nodes were classified into four categories: peripherals, connectors, module hubs and network hubs. From ecological perspectives, peripherals might represent specialists whereas module hubs and connectors were close to generalists and network hubs as super-generalists [55]. Here, the majority of OTUs (90.9 %) under warming and unwarming conditions were peripherals with most of their links inside their own modules. A total of 26 nodes (7.9 %) were connectors and only four nodes (1.2 %) were module hubs. Those four OTUs as module hubs were derived from Planctomyces (Planctomycetes), Nocardioides (Actinobacteria) under warming condition, and Thermoleophilum (Actinobacteria) and GP4 (Acidobacteria) under unwarming condition, indicating that the hubs of pMENs were substantially different under different conditions.

### The correlations between network topologies with environmental traits

The relationships between microbial network topology and environmental characteristics can be examined in both direct and indirect ways. Indirectly, as a first step, the OTU significance (*GS*) is calculated and defined as the square of Pearson correlation coefficient (*r*^2^) of OTU abundance profile with environmental traits. Then the correlation between *GS* and nodes’ topological indices (e.g., connectivity) was used to measure the relationship of network topology with traits. For instance, in warming pMEN, the *GS* of average soil temperature was significantly correlated with the nodes’ connectivity (*r* = 0.30, *p* = 4.7 × 10^-5^), indicating that the nodes with higher connectivity were inclined to have closer relationships with temperature. If multiple *GS* was involved, Mantel and partial Mantel tests could be implemented to calculate correlations between the connectivity and multiple *GS* of environmental traits to reveal the internal associations between network topology and environmental changes. In warming pMEN, the nodes’ connectivity was significantly associated with the *GS* of pH values, soil NO_3_-nitrogen and soil carbon contents when the effect of temperature was controlled (r_M_ = 0.104, P = 0.018). Meanwhile, the *GS* of temperature was also significantly associated with the connectivity when aforementioned soil geochemistry factors were controlled (r_M_ = 0.159, P = 0.003) (Table 4). Moreover, the OTUs of *β-Proteobacteria* and *Verrucomicrobia* were highly associated with the changes of soil geochemistry (r_M_ = 0.59 and 0.926 respectively, both P = 0.013). These results suggested that the OTUs topology in warming pMEN was significantly associated with both temperature and the selected soil variables. In addition, OTUs from *β-Proteobacteria* and *Verrucomicrobia* were most sensitive to the changes of soil variables.

**Table 4:** The partial Mantel tests on connectivity vs. the OTU significances of soil geochemical variables and soil temperature in warming pyrosquencing molecular ecological network

**Phylogeny**	**# nodes**	***GS*of soil geochemistry**^**a**^**partial*GS*of temperature**		***GS*of temperature partial*GS*of soil geochemistry**	
		**r**_**M**_^**b**^	**P**^**c**^	**r**_**M**_	**P**
All detected OTUs	177	0.104	**0.018**	0.159	**0.003**
*Acidobacteria*	35	0.059	0.234	−0.054	0.800
*Actinobacteria*	63	−0.033	0.650	0.077	0.135
*Chloroflexi*	5	−0.339	0.663	0.367	0.108
*Planctomycetacia*	6	−0.082	0.521	−0.202	0.788
*α-Proteobacteria*	26	−0.057	0.721	0.096	0.155
*β-Proteobacteria*	12	0.590	**0.013**	−0.001	0.430
*δ-Proteobacteria*	6	0.338	0.088	−0.298	0.877
*γ-Proteobacteria*	4	0.030	0.772	0.796	0.243
*Verrucomiobia*	5	0.926	**0.013**	−0.755	1.000

^a^Soil variables used for OTU significance calculations: pH values, NO_3_-Nitrogen and soil carbon contents.

^b^Correlation coefficient based on Mantel test.

^c^The significance (probability) of Mantel test.

**Figure 5:**
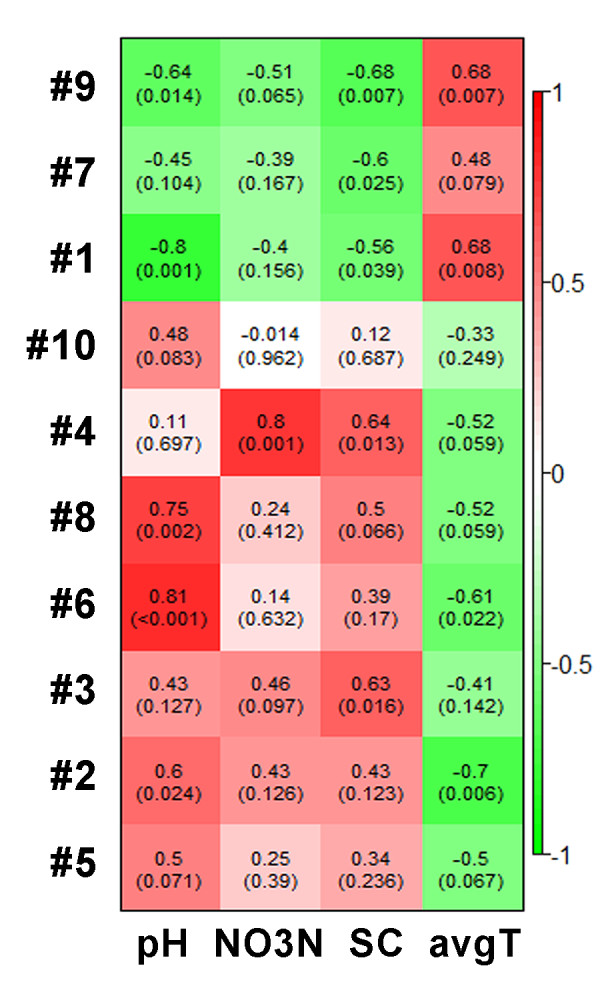
**The correlations between module eigengenes and environmental traits in the warming pMEN.** The color of each plot indicates the correlation between corresponding module eigengene and environmental trait. Red color means highly positive correlation and green color means highly negative correlation. The numbers in each plot are the correlation coefficient (*r*) and significance (*p*) in parentheses. The environmental traits include soil pH value (pH), NO_3_-nitrogen content (NO_3_N), soil carbon content (SC) and average soil temperature (avgT).

The correlations between module-based eigengenes and environmental factors can be used to detect the modules’ response to environmental changes. In warming pMEN, the coefficients (*r* values) and significances (*p* values) were shown in a heatmap (Figure 5). Submodules #1 and #9 were positively correlated with the average soil temperature significantly (*p* < 0.01) but negatively (*p* < 0.01) with soil pH values and soil carbon contents, indicating that the members in these two submodules might be stimulated by temperature but inhibited by soil pH and carbon. Also, submodules #6 and #8 were positively correlated with soil pH (*p* < 0.01), #4 was positively correlated with NO_3_^-^ concentration (*p* = 0.001) and soil carbon content (*p* = 0.013). While #3 was positively correlated with carbon content (*p* = 0.016), #7 was negatively correlated with soil carbon content (*p* = 0.025). In addition, #2 and #6 were negatively correlated with temperature (p < 0.05). All above results demonstrated that different submodules in warming pMEN responded to the environmental changes differently and the changes of temperature could have significant impacts on members of some submodules (e.g., #1, #2, #6 and #9).

### Open-access pipeline

**Figure 6:**
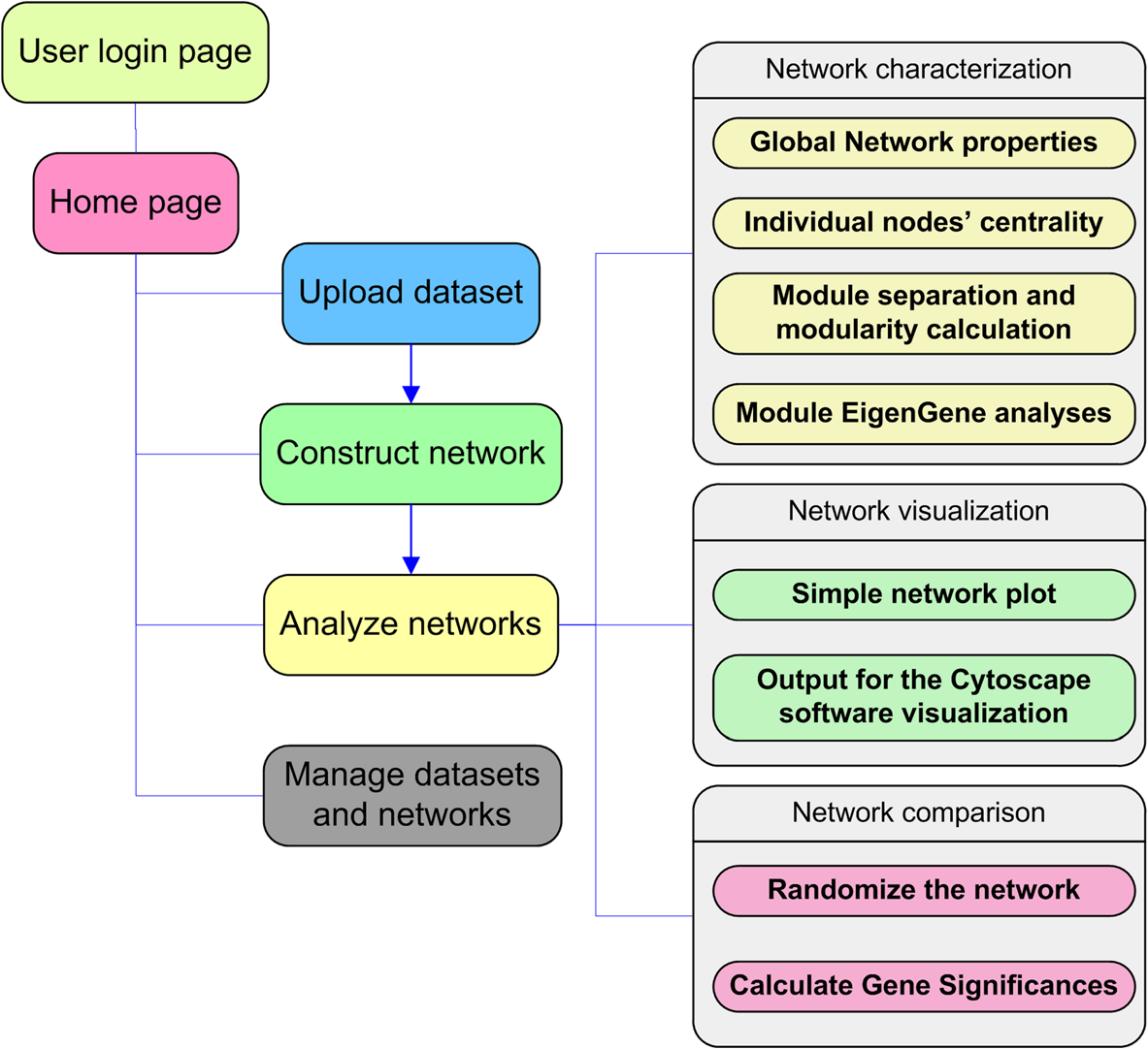
An overview of molecular ecological network analysis pipeline (MENAP).

To facilitate the application of MENA in the scientific community, an open-access pipeline for MEN construction and analysis (MENAP) was implemented (http://ieg2.ou.edu/MENA). Although currently microarray-based intensity data and pyrosequencing data are two major types of informational sources for microbial community network analysis, a variety of other data types can be used for this pipeline as well. MENAP is implemented in Perl integrated common gateway interface (CGI) and runs on a Windows Server (Windows Server 2007). A user-friendly interface through web browser application was developed to facilitate the process of RMT-based network construction and related analyses (Figure 6). RMT-based threshold searching is performed using a Java script [22] and some network analyses are called in the programs of sna [63], igraph [64] and WGCNA [65] packages in the R project. The MENAP includes the following components: (i) user registration and login, (ii) data upload, (iii) network construction by the RMT-based method (perhaps other methods as well), (iv) network analysis, and (v) dataset and network management (Figure 6).

The network analysis component is further divided into three major parts:

(a) Network characterization. Various network properties are calculated and evaluated, such as connectivity, betweenness, clustering coefficient, and geodesic distance. The module/submodule detection and modularity analyses is performed using fast greedy modularity optimization [66]. Eigengene network analysis is performed to understand network characteristics at higher organization levels and to identify key microbial populations or key functional genes in terms of network topology.

(b) Network visualization. An automatic pipeline is constructed to visualize the constructed network. Moreover, the file format for software Cytoscape 2.6.0 [67] is prepared to visualize more complex and delicate network graphs. Other data associated with OTUs, such as taxonomy, relative abundance, edge information, and positive and negative correlations is imported and visualized in network figures.

(c) Network comparison. Various randomization methods like the Maslov-Sneppen method [68] are used obtain random networks for network comparison. Various indices are evaluated for comparing the differences of networks among different communities in terms of sensitivity and robustness. In addition, OTU significances are calculated to reveal associations of the network structure to the ecological functional traits [27].

## Discussion and conclusions

Most previous studies on the biodiversity of microbial communities have been focused on the number of species and the abundance of species, but not interactions among species. However, species interactions could be more important to ecosystem functioning than species richness and abundance, especially in complex ecosystems [1,27-29]. Several recent analyses show that the ecological networks of ecosystems are highly structured [1,69,70], thus ignoring the structure of network and the interactions among network components precludes further assessment of biodiversity and its dynamics. Several recent breakthroughs have been made to analyze species interactions of animals and plants [1,4,31,55,70,71], but it is difficult to detect network interactions of a microbial community [72-74]. Therefore, in this study, we systematically described a mathematical and bioinformatic framework of MENA based on RMT, a powerful method well established in quantitative physics [23,75,76]. Our results demonstrate that the RMT-based approach is powerful in discerning network interactions in microbial communities.

The network approach described is based on the transition of two universal distributions from the random matrix theory. A major advantage of RMT method is that the threshold to construct network is automatically determined. In contrast, most other methods studies use arbitrary thresholds, which are usually based on limited knowledge of biological information [8,72-74,77]. RMT-based approach selects an optimal threshold without ambiguity, which ensures its construction of optimal networks. Another advantage of RMT-based approach is its remarkable capacity in tolerating noise, resulting in reliable, robust networks. Our results show even with 100 % Gaussian noise, more than 85 % nodes from original network are still preserved. This characteristic could be very important for dealing with the large-scale data, such as metogenomics and micrrorrays, which are generally inherent with high noise.

Nevertheless, characterizing ecological network of microbial communities poses major challenges. MENs are constructed by the adjacency matrix originated from the pair-wise correlations of relative OTU abundance across different samples. Therefore, a network interaction between two OTUs or genes describes the co-occurrence of these two OTUs or genes across different samples. The co-occurrence might be caused by species or genes performing similar or complementary functions, or shared environmental conditions that microbial species coexist in [28]. However, the former possibility can be complicated by the observations that functionally redundant genes are not necessary co-regulated, but instead co-regulated through other genes, which is coined as transitive co-regulation [78]. The latter possibility can be complicated by the distinctiveness of individuals in microbial niches observed in their behaviors and responses to environmental perturbation [79]. Therefore, caution must be taken for the interpretation of underlying mechanisms that shape microbial communities.

A long-held tenet is that the structure of ecological networks has significant influence on the dynamics [1,80]. Most complex systems have common characteristics such as small world, scale-free, modularity and hierarchy [8,53,54]. Consistently, MENs were found to be scale-free, small world and modular, in addition to hierarchical property in some MENs. These network properties are important for the robustness and stability of complex systems [8,27,28,81]. For example, our results showed that any two microbial species in the community can be linked by just a few other neighbor species, showing small-world property. This may imply that the energy, materials and information can be easily transported through entire systems. In microbial communities, this characteristic drives efficient communications among different members so that relevant responses can be taken rapidly to environmental changes. Meanwhile, it is intriguing to note that modularity is prevailing in MENs, while hierarchy is present only in some MENs. Research on a wide range of architectural patterns in mutualistic (pollination) and trophic (predation) networks showed that hierarchy, also called nestedness, was strong in mutualistic networks, but that modularity was strong in trophic networks [82]. Although ecological networks of microbial communities are very complicated and cannot be classified into simple mutualistic or trophic networks, it would be interesting to compare a number of ecological networks of microbial communities to catalog different architectural patterns and to explore the mechanisms underlying the stability and resilience of communities.

In addition to interactions among microbes within a community, MENs allow for analyses of interactions with their environment through correlations with abiotic environmental measurements, which might provide insights on the conditions that have significant impact on the co-occurring organisms. It is also possible to link groups of organisms with biogeochemical measurements to reveal the functional role of organism in biogeochemical processes. These kinds of data are important for generating hypotheses to help explain natural environments that microbial communities reside, which might lead to forecasting responses of microbial communities when environment changes [73].

In summary, our study provides a mathematical/bioinformatic framework for network construction based on metagenomics data such as sequencing [28] and microarray hybridization data [27]. It is useful, as demonstrated with the microbial communities under experimental warming, for dissecting interactions within a microbial community as well as with environment, thus allowing microbial ecologists to address a variety of ecological questions at the community-wide scale [83,84]. It is also possible to extend MENA to emerging fields of microbial ecology such as high-throughput proteomics, since RMT is not stringent on data types. In addition, broad application of MENA will generate a number of ecological networks that allow for exploration of architectural patterns of microbial communities [1]. This RMT-based molecular ecological network analysis provides powerful tools to elucidate network interactions in microbial communities and their responses to environmental changes, which are fundamentally important for research in microbial ecology, systems microbiology, and global change.

## Methods

### Data standardization

The network construction begins with a data table with *n* distinct operational taxonomic units (OTUs) based on 16 S rRNA genes or functional genes observed across *m* replicates or samples. Typically OTUs are used to refer taxonomic classification based on ribosomal RNA genes. For convenience, in the following sections, we use OTUs to refer the classifications derived from both 16 S rRNA genes and/or functional genes. Let *y*_*ik*_ represent the abundance or relative abundance of the *i-*th OTU in the *k-*th sample (i∈{1,…,n}, k∈{1,…,m}) and *Y*^nxm^ = [*y*_*ik*_] is the abundance matrix. Usually, the abundance profile of *i*-th OTU is standardized as below. If the mean and standard deviation of *y*_*i*_ across all samples are ${\bar{y}}_{i}$and $\sigma_{i}$, the standardized abundance of the *i-*th OTU in the *k-*th sample is $x_{\mathrm{ik}}=\frac{\left(y_{\mathrm{ik}}-{\bar{y}}_{i}\right)}{\sigma_{i}}$, where *x*_*ik*_ has mean value of 0 and variance value of 1. *X*^nxm^ is the standardized data matrix and used for subsequent correlation analysis.

### Defining adjacency matrix

Molecular ecological networks can be built on the basis of the measurements of relative OTU abundance in microbial communities. In MENs, each OTU corresponds to a node. Each network corresponds to an adjacency matrix (or interaction matrix), *A*^nxn^ = *a*_*ij*_, which encodes the connection strength between each pair of nodes [20]. In an unweighted network, the adjacency *a*_*ij*_ =1 if nodes *i* and *j* are connected, and *a*_*ij*_ =0 otherwise [20]. For an undirected network, the adjacency matrix is symmetric. In weighted network, the pairwise adjacency has values between 0 and 1, i.e., 0 ≤ *a*_*ij*_ ≤ 1. The adjacency matrix is the foundation of all subsequent steps in network analysis.

To define the adjacency matrix, the similarity of OTU abundance across all samples should be measured first. Such similarity measures the degree of concordance between the abundance profiles of OTUs across different samples. Similar to widely used gene co-expression analyses [20,61,85,86], Pairwise Pearson correlation coefficients (*r*_*ij*_) are used to measure the similarity between *i-*th and *j-*th OTU across different samples. Let *R*^nxn^ = *r*_*ij*_ be the Pearson correlation matrix, then

(1) $$r_{\mathrm{ij}}=cor(x_{i},x_{j})=\frac{\sum_{k=1}^{m}(x_{\mathrm{ik}}-{\bar{x}}_{i})(x_{\mathrm{jk}}-{\bar{x}}_{j})}{\sqrt{\sum_{k=1}^{m}{\left(x_{\mathrm{ik}}-{\bar{x}}_{i}\right)}^{2}\sum_{k=1}^{m}{\left(x_{\mathrm{jk}}-{\bar{x}}_{j}\right)}^{2}}}$$

where *x*_*ik*_ and *x*_*jk*_ are the standardized abundance of the *i*-th and *j*-th OTUs in the *k*-th sample. ${\bar{x}}_{i}$${\bar{x}}_{j}$ are the mean values of the *i*-th and *j*-th OTUs over samples. In general, the absolute value of the correlation coefficient (*r*_*ij*_) is used to define the abundance similarity between *i-*th and *j-*th OTU (*s*_*ij*_), that is

(2) $$s_{\mathrm{ij}}=\left|r_{\mathrm{ij}}\right|,where\;i\;j\in \{1,\ldots ,n\}$$

Let *S*^nxn^ = [s_ij_, which is a similarity matrix of the OTU abundance. In molecular ecological network analysis, the adjacency matrix is derived from the OTU abundance similarity matrix by applying a threshold. Similar to relevant gene co-expression network analysis [20,61,85,86], the nodes are connected if they have significant pairwise similarities (i.e., correlations) across different samples. Thus, using a threshold value (*s*_*tb*_), OTU abundance similarity matrix, *S*^n×n^ = *s*_*ij*_, is converted into the adjacency matrix, *A*^p×p^ = *a*_*ij*_, where p ≤ n. The adjacency *a*_*ij*_ between the *i-*th and *j-*th OTU is defined by thresholding the OTU abundance similarity [33]:

(3) $$a_{\mathrm{ij}}=\{\begin{array}{c} s_{\mathrm{ij}}\;if\;s_{\mathrm{ij}}\geq s_{\mathrm{tb}}\\ 0\;if\;s_{\mathrm{ij}}<s_{\mathrm{tb}} \end{array}$$

where *s*_*tb*_ is the threshold parameter. The resulting adjacency matrix, *A*^p×p^, is generally smaller than the similarity matrix because the rows or columns are removed if all of their elements are less than the threshold value.

### Determining the threshold by random matrix theory-based approach

The structure of relevance network strongly depends on the threshold value, *s*_*t*_. In some network analysis, the threshold value is chosen arbitrarily based on known biological information or set by the empirical study [8]. Thus, the resulting network is more or less subjective [19,20,85,87]. However, it is difficult to select appropriate thresholds, especially for poorly studied organisms/communities. In MENA, we use the random matrix theory (RMT)-based approach, which is able to identify the threshold automatically based on the data structure itself [22,46] to select the final threshold parameter, *s*_*t*_.

### Basic concept of RMT

Initially proposed by Wigner and Dyson in the 1960s for studying the spectrum of complex nuclei [88], random matrix theory (RMT) is a powerful approach for identifying and modeling phase transitions associated with disorder and noise in statistical physics and materials science. It has been successfully used for studying the behavior of different complex systems, such as spectra of large atoms [89], metal insulator transitions in disorder systems, spectra of quasiperiodic systems [90], chaotic systems [91], the stock market [76], brain response [92], gene co-expression networks [22] and protein interaction networks [46]. However, its suitability for complex biological systems, especially microbial communities, remains largely unexplored.

RMT predicts two universal extreme distributions of the nearest neighbor spacing distribution (NNSD) of eigenvalues: Gaussian orthogonal ensemble (GOE) statistics, which corresponds to random properties of complex system, and Poisson distribution, which corresponds to system-specific, nonrandom properties of complex systems [89]. These two different universal laws depend on the properties of the matrix. On one hand, if consecutive eigenvalues are completely uncorrelated, the NNSD follows Poisson statistics. Considering a series of eigenvalues, the probability of an eigenvalue falling in a scale *DD* + *s* is independent of the start point *D*, where *s* can be any positive values. It means the probability of an eigenvalue falling in any scales with certain length *s* will be identical, no matter where the scales begin. The NNSD under such assumption follows a Poisson random process, so-called exponential distribution of Poisson process [89]. On the other hand, for correlated eigenvalues, the NNSD has Gaussian orthogonal ensemble (GOE) statistics. Given a series of correlated eigenvalues, the probability of one eigenvalue falling into a scale *DD* + *s* is proportional to *s*. Wigner illustrated that the NNSD under this assumption was closely to Gaussian orthogonal ensemble so-called *Wigner surmise*[89].

The key concept of RMT is to mainly concern with the *local property* between eigenvalues rather than the *global property* of a series of eigenvalues. Here, the local property between eigenvalues means the eigenvalue fluctuations and the global property is the average eigenvalue density. In order to reveal the fluctuations of eigenvalues, the average eigenvalue density has to be removed from system so that the average eigenspacing is constant. Also, this procedure to generate a uniform eigenvalues distribution is called *unfolding*. The unfolded eigenvalues will fall between 0 and 1, and its density does not depend on the overall level distribution. Consider a sequence of eigenvalues ${\{}_{\lambda 1}{,}_{\lambda 2}{,}_{\mathrm{\ldots \lambda}n}\}$ from adjacency matrix, and those eigenvalues have been ordered as ${\text{\{λ}}_{1}\leq {\text{λ}}_{2}\leq \ldots \leq \lambda_{n}\}$. In practice, we replace eigenvalues *λ*_*i*_ with$e_{i}=N_{\mathrm{av}}(\lambda_{i})$ where *N*_*av*_ is the continuous density of eigenvalues obtained by fitting and smoothing the original integrated density of eigenvalues to a cubic spline or by local density average.

After unfolding the eigenvalues, three statistical quantities can be used to extract information from a sequence of eigenvalues, namely, eignevalue spacing distribution *P*(*d*), number variance of eigenvalues ∑, and spectral rigid ▵. *P*(*d*) is the probability density function for unfolded eigenvalue spacing, $d_{i}=\left|e_{i+1}-e_{i}\right|$, which is the NNSD for eigenvalues. For the completely uncorrelated eigenvalues, *P*(*d*) follows Poisson statistic and it can be expressed by

(4) P(d)=exp(−d).

On the other hand, for the correlated eigenvalues, *P*(*d*) closely follows Wigner-Dyson distribution of the GOE statistics and it can be expressed by

(5) $$P(d)\approx \frac{\pi d}{2}\exp(-\frac{\pi }{2}d^{2})\text{.}$$

We use the *χ*^2^ goodness-of-fit test to assess whether NNSD follows Wigner-Dyson distribution or Poisson distribution. We assume that the NNSD of any biological system obeys these two extreme distributions [22,23,27,28], and that there is a transition point from GOE to Poisson distribution, and this transition point can be used as the threshold for defining adjacency matrix.

### Algorithms of detecting the threshold value

The following major steps are used to define the threshold (*s*_*t*_) based on the standardized relative abundance of OTUs across different samples (Figure 2).

(a) Calculate the Pearson correlation matrix, *R*^nxn^, based on the standardized relative abundance of OTUs, *X*^nxm^ with *n* distinct OTUs across *m* samples.

(b) Obtain similarity data, *S*^nxn^, by taking the absolute value of correlation matrix *R*^*n×n*^.

(c) Set an initial threshold value, *s*_*tb*_ (e.g., 0.3 based on our experiences).

(d) Calculate the adjacency matrix, *A*^pxp^ = [*a*_*ij*_] according to *s*_*tb*_, where *p* is the number of OTUs retained in the adjacency matrix with non-zero rows or columns.

(e) Calculate eigenvalues *λ*_*i*_ of the adjacency matrix based on the equation(S−λI)v=0, where *λ* is the eigenvalue, *v* is the corresponding eigenvector, and *I* is the identity matrix. Because *S* is the symmetric matrix and *v* is a non-zero vector, we can get *p* number of eigenvalues to solve the equation(S−λI)v=0. To test NNSD distribution, order the eigenvalues as$\lambda_{1}\leq \lambda_{2}\leq \ldots \leq \lambda_{\text{p}}\text{.}$

(f) To get unfolded eigenvalues, replace *λ*_*i*_ with$e_{i}=N_{\mathrm{av}}(\lambda_{i})$, where *N*_*av*_ is the continuous density of eigenvalues and can be obtained by fitting the original integrated density to a cubic spline or by local average.

(g) Calculate the nearest neighbor spacing distribution of eigenvalues, *P*(*d*), which defines the probability density of unfolded eigenvalues spacing, $d_{i}=\left|e_{i+1}-e_{i}\right|\text{.}$

(h) Using the *χ*^2^ goodness-of-fit test to determine whether the probability density function *P*(*d*) follows the exponential distribution of Poisson statistic, exp(−d).H_0_: *P*(*d*) follows the Poisson distribution.H_1_: *P*(*d*) does not follows the Poisson distribution.The *χ*^2^ goodness-of-fit test has the test statistics, $\chi^{2}=\sum_{i}\frac{{\left[d_{i}-E(d_{i})\right]}^{2}}{E(d_{i})}$, where *d*_*i*_ is the observed nearest neighbor spacing and *E*(*d*_*i*_) is an expected (theoretical) nearest neighbor spacing from Poisson distribution. The resulting *χ*^2^ value is compared to the *χ*^2^ distribution. Let $\chi_{u}{}^{2}(0.01)$be the critical value at a significant level of 0.01 based on *χ*^2^ distribution with *u* degrees of freedom.

(i) If $\chi^{2}\leq \chi_{u}{}^{2}(0.01)$, the null hypothesis H_0_ is not rejected. Then go to step (j).If $\chi^{2}>\chi_{u}{}^{2}(0.01)$, the null hypothesis H_0_ is rejected. Then, increase the threshold by 0.1, *s*_*tb*_ + 0.1, and repeat the steps from (e) to (h).

(j) Find a finer scale threshold value by increasing the threshold with 0.01 within the range of [*s*_*tb*_-0.1, *s*_*tb*_]. Then repeat the steps from (e) to (h).

(k) If H_0_ is accepted, i.e., the *P*(*d*) follows Poisson distribution, the finer scale threshold identified is used as the optimal threshold for defining the adjacency matrix.

Once the final threshold value *s*_*t*_ is determined at a finer scale, an adjacency matrix is obtained by retaining all the OTUs whose abundance similarity values are greater than the determined threshold. Currently we have only adopted the unweighted network in the following network topological analysis. Hence, the final adjacency *a*_*ij*_ is:

(6) $$a_{\mathrm{ij}}=\{\begin{array}{c} 1\;if\;s_{\mathrm{ij}}\geq s_{t}\\ 0\;if\;s_{\mathrm{ij}}<s_{t} \end{array}\text{.}$$

where *s*_*t*_ is the final threshold parameter. Two nodes are linked if the similarity between their abundance profiles across all samples is equal to 1.

### Calculation of MEN topological indices and general features

Once MENs are determined, various network topology indices can be calculated based on the adjacency matrix (Table 1). The overall topological indices describe the overall network topology in different views and thus are useful in characterizing various MENs identified under different situations. The indices for describing individual nodes are useful in assessing their roles in the network.

Scale-free, small world, modularity and hierarchy are most common network characteristics of interest [8,53,93]. A scale-free network is a network whose connectivity follows a power law, at least asymptotically [94], that is, only a few nodes in the network have many connections with other nodes while most of nodes have only a few connections with other nodes. It can be expressed by $P(k)\sim k^{-\lambda }$, where *k* is connectivity and *λ* is a constant. A small-world network is the network in which most nodes are not neighbors of one another, but most nodes can be reached by a few paths (typically, less than 6). Small world network has a small average shortest path (*GD*) typically as the logarithm of the number of nodes [43]. In addition, there is no formal definition for hierarchical topology [95]. One of the most important signatures for hierarchical, modular organizations is that the scaling of clustering coefficient follows *C*(*k*) ~ *k*^−*γ*^, in which *k* is connectivity and *γ* is a constant. By log-transformation, log*C(k)* ~ −*γ*log(*k*), the logarithms of clustering coefficients have a linear relationship with the logarithms of connectivity.

### Module detection

Modularity is a fundamental characteristics of biological networks as well as many engineering systems [53]. In MENs, a module in the network is a group of OTUs that are highly connected within the group, but very few connections outside the group. The maximum modularity score is used to separate the graph into multiple dense sub-graphs or modules. The modularity of each network (*M*) is estimated using the equation [66]:

(7) $$M=\sum_{b=1}^{N_{M}}\left[\frac{l_{b}}{L}-{\left(\frac{K_{b}}{2L}\right)}^{2}\right]\text{,}$$

where *N*_*M*_ is the number of modules in the network, *l*_*b*_ is the number of links among all nodes within the *b*^th^ module, *L* is the number of all links in the network, and $K_{b}$ is the sum of degrees (connectivity) of nodes which are in the *b*^th^ module. *M* measures the extension whose nodes have more links within their own modules than expected if linkage is random. It varies with the range of [−1, 1].

Several different algorithms can be used to separate modules, including short random walks, leading eigenvector of the community matrix, simulated annealing approach, and fast greedy modularity optimization [56,57]. The algorithm of short random walks is based on the idea that all random walks tend to stay in the densely connected parts of a graph that was corresponding to the modules [56]. After calculating a distance between two nodes or between sets of nodes by random walk algorithm, it uses a hierarchical clustering approach to present the structural similarities between all nodes. Thereafter this approach will choose the best partition automatically. The advantage of this algorithm is efficient and fast computation.

Once the network modularity value (*M*) was explicitly defined, theoretically the module structure can be determined by maximizing *M* values over all possible divisions of network. However, exhaustive maximization over all divisions is computational intractable [57]. The algorithm of leading eigenvector is one of several approximate optimization methods have been proven effectively obtained higher *M* values with high speed. It simplified the maximization process in terms of a modularity matrix B^nxn^ that can be obtained by the adjacent matrix A^nxn^ subtracting an expected edges matrix P^nxn^ from a null model. Then the network can be split into two groups by finding the leading eigenvector that was corresponding to the largest positive eigenvalue of modularity matrix. This splitting process can be looped until any further divisions will not increase the *M* value [57]. This method shows more accurate separations than other algorithms in several well-studied social networks [57].

The algorithm of simulated annealing approach usually produces the best separation of the modules by direct maximization of *M*[58]. The simulated annealing is a stochastic optimization technique to find “low cost” configurations [96]. It carries out the exhaustive search on network structures to merge and split *priori*-modules and move individual nodes from one module to another. Although this is a time-consuming process, it is expected to obtain clear module separations with a higher *M*.

The algorithm of fast greedy modularity optimization is to isolate modules via directly optimizing the *M* score [66,97]. It starts with treating each node as the unique member of one module, and then repeatedly combines two modules if they generate the largest increase in modularity *M*. This algorithm has advantages with fast speed, accurate separations and ability to handle huge networks [66,97].

### Identification of key module members

After all modules are separated, each node can be assigned a role based on its topological properties [59], and the role of node *i* is characterized by its within-module connectivity (*z*_*i*_) and among-module connectivity (*P*_*i*_) as follows

(8) $$z_{i}=\frac{k_{\mathrm{ib}}-{\bar{k}}_{b}}{\sigma_{k_{b}}}\text{,}$$

and

(9) $$P_{i}=1-\sum_{c=1}^{N_{M}}{\left(\frac{k_{\mathrm{ic}}}{k_{i}}\right)}^{2}\text{,}$$

where $k_{\mathrm{ib}}$is the number of links of node *i* to other nodes in its module *b*${\bar{k}}_{b}$ and $\sigma_{k_{b}}$are the average and standard deviation of within-module connectivity, respectively over all the nodes in module *b,*$k_{i}$ is the number of links of node *i* in the whole network, $k_{\mathrm{ic}}$ is the number of links from node *i* to nodes in module *c*, and *N*_*M*_ is the number of modules in the network.

The within-module connectivity, *z*_*i*_, describes how well node *i* is connected to other nodes in the same module, and the participation coefficient, *P*_*i*_, reflects what degree that node *i* connects to different modules. *P*_*i*_ is also referred as the among-module connectivity [98]. If all links of node *i* only belong to its own module, *P*_*i*_ = 0. If the links of node *i* are distributed evenly among modules, *P*_*i*_ → 1. The topological roles of individual nodes can be assigned by their position in the *z*-parameter space. Originally, Guimera et al. [33,59] divided the topological roles of individual nodes into seven categories. Olesen et al. [98] simplified this classification into four categories for pollination networks. In this study, we use the simplified classification as follows: (i) Peripheral nodes (*z*_*i*_ ≤ 2.5, *P*_*i*_ ≤ 0.62), which have only a few links and almost always to the nodes within their modules, (ii) Connectors (*z*_*i*_ ≤ 2.5, *P*_*i*_ > 0.62), which are highly linked to several modules, (iii) Module hubs (*z*_*i*_ > 2.5, *P*_*i*_ ≤ 0.62), which are highly connected to many nodes in their own modules, and (iv) Network hubs (*z*_*i*_ > 2.5, *P*_*i*_ > 0.62), which act as both module hubs and connectors. From ecological perspective, peripheral nodes represent specialists whereas the other three are generalists.

### Eigen-gene analysis

One of the grand challenges in dealing with high throughput metagenomics data is the high dimensionality. Various statistical approaches are used to reduce dimensions and extract major features, including principal component analysis (PCA), detrended correspondence analysis (DCA), and singular value decomposition (SVD). SVD is an orthogonal linear transformation of data (e.g., microbial data) from the complexity to the comprehensibility [99]. Based on SVD analysis, the Eigengene is a linear combination of genes and eigenvalues. In the diagonalized data, each eigengene is just expressed in the corresponding eigen arrays. Langfelder and Horvath [61] proposed eigengene network analysis to summarize the gene expression data from each module as a centroid. Eigengene network analysis is powerful to reveal higher order organization among gene co-expression modules [33,61,62]. Here, we have adopt this method to analyze modules in MENs.

#### *SVD analysis to define module eigen-gene*

Suppose there are *n*^*b*^ OTUs in the *b*-th module. Let $X^{b}=[x_{i,q}^{b}]$ represent the relative abundance matrix of the *b*-th module, where $x_{i,q}^{b}$is the relative abundance of the *i*-th OTU in the *q*-the sample ($i\in \{1,\ldots ,n^{b}\}$, q∈{1,…,m}). In SVD analysis, *X*^*b*^ can be decomposed as follows:

(10) $$X^{b}=U^{b}D^{b}{\left(V^{b}\right)}^{T}\text{,}$$

where both $U^{b}{}_{\left(n^{b}\times m\right)}$ and $V^{b}{}_{\left(m\times m\right)}$ are column-orthogonal matrices, and $D^{b}{}_{\left(m\times m\right)}$ is a diagonal matrix of the singular values $\left\{\left|d_{q}^{b}\right|\right\}$. The matrices *V*^*b*^ and *D*^*b*^ are denoted as$V^{b}=(v_{1}^{b},v_{2}^{b},\ldots v_{m}^{b})$ and $D^{b}=diag(|d_{1}^{b}|,|d_{2}^{b}|,\ldots ,|d_{m}^{b}|)\text{.}$

Assuming that the singular values are arranged in decreasing order, the first column of *V*^*b*^ is referred as the Module Eigen-gene, *E*^*b*^, for the *b*-th module. That is, $E^{b}\equiv v_{1}^{b}$_._

The relative abundance profile of the OTUs within a module is represented by the eigen-gene. In addition, the sum of variance of OTU abundances equals to the sum of the diagonal matrix in SVD. Therefore, the percentage of the variance explained by the eigen-gene is given by $\Phi^{b}$ as

(11) $$\Phi^{b}=\frac{|d_{1}^{b}{|}^{2}}{\sum_{j=1}^{m}|d_{j}^{b}{|}^{2}}.$$

Generally, the module eigen-gene can explain approximately 50 % or more of the variance of the OTU abundances in the module [61]. Since PCA and SVD are identical if each OTU relative abundance has been standardized to mean 0 and variance 1, *E*^*b*^ is the first principal component based on PCA analysis [61].

#### Module membership

Module eigen-gene provides the best summary of variation in relative abundance of OTUs within a module, but it is a centroid of a module rather than a real OTU. In practice, it is always important to understand how close it is between a given actual OTU and its eigen-gene. The correlation of the eigen-gene in module *b* to the *i*-th actual OTU across all experimental samples is defined as

(12) $$MM_{i}^{E^{b}}=cor(x_{i},E^{b})$$

If $M\;M_{t}^{E^{\text{b}}}$ is close to 1 or −1, it is evident that the *i*-th OTU is close to the centroid of module *b*.

### Random network construction and network comparison

Since only a single data point is available for each network parameter, we are not able to perform standard statistical analyses to assess statistical significances. Similar to the concept of hypothesis testing, the null model is generated to assess the performance of the alternative model. Thus, the random networks are generated to compare different complex networks using the Maslov-Sneppen procedure [68]. The Maslov-Sneppen method keeps the numbers of nodes and links unchanged but rewires the positions of all links in the MENs so that the sizes of networks are the same and the random rewired networks are comparable with original ones. This method has been typically used for ecological network analyses [4]. For each network identified, a total of 100 randomly rewired networks are usually generated by the Maslov-Sneppen procedure [68] and all network indices are calculated individually for each randomized network. Then the average and standard deviation for each index of all random networks are obtained. The statistical *Z*-test is able to test the differences of the indices between the MEN and random networks. Meanwhile, for the comparisons between the network indices under different conditions, the Student *t*-test can be employed by the standard deviations derived from corresponding random networks.

### Trait-based gene significance measure

In gene expression network analyses, the gene significance (*GS*_*i,h*_) is the correlation between the expression profile of the *i*-th gene and the *h*-th sample trait, *T*_*h*_[33]. The higher *GS*_*i,h*_, the more biologically significant gene *i* is related to the sample trait *h*. Similarly, in this study, the trait-based OTU significance is defined as:

(13) $$GS_{i,h}={\left[cor(x_{i},T_{h})\right]}^{2}$$

where *x*_*i*_ is the relative abundance of the *i-*th OTU i∈{1,…,n} and *T*_*h*_ is the *h-*th sample trait (e.g. soil pH, N content, total plant biomass) (h∈{1,…,g}). Since the measurement units for different traits vary, all trait data should be standardized prior to statistical analysis. Finally, an OTU significance matrix, *GS*^nxg^, is obtained.

### Relationships of microbial interaction networks with soil variables

To discern the relationships between molecular ecological networks and soil properties, Mantel tests can be performed [100]. The relationships between the MENs and environmental variables were determined as follows: First, the significances of variables are calculated with the above equation (Eq 13) and the OTU significance matrix is generated. Then the Euclidean distance matrix $D_{\mathrm{GS}}^{n\times n}$ is generated by calculating the Euclidean distance between every two OTUs. The distance matrix among all OTUs’ connectivity ($D_{k}^{n\times n}$) was calculated as well. In addition, Mantel tests are performed between the distance matrices of the connectivity ($D_{\mathrm{GS}}^{n\times n}$) and OTU significance ($D_{\mathrm{GS}}^{n\times n}$) to examine the relationships between network structure (i.e., connectivity) and soil variables. The Mantel tests were performed using the programs available in R vegan package [101].

## Competing interests

The authors declare that they have no competing interests.

## Authors’ contributions

YD carried out the analysis, constructed the pipeline and wrote the article. YJ carried out part of method development and method writing. YY provided the RMT method and contributed part of the discussion. ZH contributed the paper writing and oversight the work. FL provided the RMT method. JZ provided oversight of the work and helped finalize the article. All authors read and approved the final manuscript.

## References

[B1] Montoya JM Pimm SL Sole RV Ecological networks and their fragility Nature 2006 442 7100 259 264 10.1038/nature04927 16855581

[B2] May RM Stability and complexity in model ecosystems 1973 Princeton University Press, Princeton, New Jersey

[B3] Pim S Food webs 1982 Chapman & Hall, London

[B4] Dunne JA Williams RJ Martinez ND Food-web structure and network theory: The role of connectance and size Proc Natl Acad Sci USA 2002 99 20 12917 12922 10.1073/pnas.192407699 12235364 PMC130560

[B5] Montoya JM Sole RV Small world patterns in food webs J Theor Biol 2002 214 3 405 412 10.1006/jtbi.2001.2460 11846598

[B6] Rezende EL Lavabre JE Guimaraes PR Jordano P Bascompte J Non-random coextinctions in phylogenetically structured mutualistic networks Nature 2007 448 7156 925 U926 10.1038/nature05956 17713534

[B7] Raes J Bork P Molecular eco-systems biology: towards an understanding of community function Nat Rev Miobiol 2008 6 9 693 699 10.1038/nrmicro1935 18587409

[B8] Barabasi AL Oltvai ZN Network biology: Understanding the cell's functional organization Nat Rev Genet 2004 5 2 101 U115 10.1038/nrg1272 14735121

[B9] Akutsu T Miyano S Kuhara S Identification of genetic networks from a small number of gene expression patterns under the Boolean network model Pac Symp Biocomput 1999 17 28 10.1142/9789814447300_0003 10380182

[B10] Gardner TS di Bernardo D Lorenz D Collins JJ Inferring genetic networks and identifying compound mode of action via expression profiling Science 2003 301 5629 102 105 10.1126/science.1081900 12843395

[B11] Liang S Fuhrman S Somogyi R Reveal, a general reverse engineering algorithm for inference of genetic network architectures Pac Symp Biocomput 1998 3 18 29 9697168

[B12] Yeung MKS Tegner J Collins JJ Reverse engineering gene networks using singular value decomposition and robust regression Proc Natl Acad Sci USA 2002 99 9 6163 6168 10.1073/pnas.092576199 11983907 PMC122920

[B13] Friedman N Linial M Nachman I Pe'er D: Using Bayesian networks to analyze expression data Journal of Computational Biology 2000 7 3–4 601 620 11108481 10.1089/106652700750050961

[B14] Gerstung M Baudis M Moch H Beerenwinkel N Quantifying cancer progression with conjunctive Bayesian networks Bioinformatics 2009 25 21 2809 2815 10.1093/bioinformatics/btp505 19692554 PMC2781752

[B15] Butte AJ Tamayo P Slonim D Golub TR Kohane IS Discovering functional relationships between RNA expression and chemotherapeutic susceptibility using relevance networks Proc Natl Acad Sci USA 2000 97 22 12182 12186 10.1073/pnas.220392197 11027309 PMC17315

[B16] Chen Y Zhu J Lum PY Yang X Pinto S MacNeil DJ Zhang C Lamb J Edwards S Sieberts SK Variations in DNA elucidate molecular networks that cause disease Nature 2008 452 7186 429 435 10.1038/nature06757 18344982 PMC2841398

[B17] Horvath S Zhang B Carlson M Lu KV Zhu S Felciano RM Laurance MF Zhao W Qi S Chen Z Analysis of oncogenic signaling networks in glioblastoma identifies ASPM as a molecular target Proc Natl Acad Sci USA 2006 103 46 17402 17407 10.1073/pnas.0608396103 17090670 PMC1635024

[B18] Oldham MC Horvath S Geschwind DH Conservation and evolution of gene coexpression networks in human and chimpanzee brains Proc Natl Acad Sci USA 2006 103 47 17973 17978 10.1073/pnas.0605938103 17101986 PMC1693857

[B19] Schmitt WA Raab RM, Stephanopoulos G: Elucidation of gene interaction networks through time-lagged correlation analysis of transiptional data Genome Res 2004 14 8 1654 1663 10.1101/gr.2439804 15289483 PMC509275

[B20] Zhang B Horvath S A general framework for weighted gene co-expression network analysis Stat Appl Genet Mol Bio 2005 4 17 10.2202/1544-6115.1128 16646834

[B21] Gardner TS Faith JJ Reverse-engineering transiption control networks Phys Life Rev 2005 2 1 65 88 10.1016/j.plrev.2005.01.001 20416858

[B22] Luo F Yang Y Zhong J Gao H Khan L Thompson DK Zhou J Constructing gene co-expression networks and predicting functions of unknown genes by random matrix theory BMC Bioinformatics 2007 8 299 10.1186/1471-2105-8-299 17697349 PMC2212665

[B23] Luo F Zhong JX Yang YF Scheuermann RH Zhou JZ Application of random matrix theory to biological networks Phys Lett A 2006 357 6 420 423 10.1016/j.physleta.2006.04.076

[B24] Yang Y Harris DP Luo F Xiong W Joachimiak M Wu L Dehal P Jacobsen J Yang Z Palumbo AV Snapshot of iron response in Shewanella oneidensis by gene network reconstruction BMC Genomics 2009 10 1 131 10.1186/1471-2164-10-131 19321007 PMC2667191

[B25] Handelsman J Tiedje JM Alvarez-Cohen L Ashburner M Cann IKO DeLong EF Doolittle WF Fraser-Liggett CM Godzik A Gordon JI Committee on Metagenomics: Challenges and Functional Applications 2007 National Academy of Sciences, Washington 1 158

[B26] He Z Gentry TJ Schadt CW Wu L Liebich J Chong SC Huang Z Wu W Gu B Jardine P GeoChip: a comprehensive mioarray for investigating biogeochemical, ecological and environmental processes ISME J 2007 1 1 67 77 10.1038/ismej.2007.2 18043615

[B27] Zhou J Deng Y Luo F He Z Tu Q Zhi X: Functional molecular ecological networks. mBio 2010 1 4 e00169 00110 10.1128/mBio.00169-10 PMC2953006 20941329

[B28] Zhou J Deng Y Luo F He Z Yang Y Phylogenetic molecular ecological network of soil miobial communities in response to elevated CO2 mBio 2011 2 4 e00122–11 21791581 10.1128/mBio.00122-11 PMC3143843

[B29] Bascompte J Networks in ecology Basic Appl Ecol 2007 8 6 485 490 10.1016/j.baae.2007.06.003

[B30] Dunne JA Williams RJ Martinez ND Wood RA Erwin DH Compilation and network analyses of cambrian food webs PLoS Biol 2008 6 4 e102 10.1371/journal.pbio.0060102 18447582 PMC2689700

[B31] Dunne JA The network structure of food webs. In: Ecological Networks: Linking Structure to Dynamics in Food Webs. Edited by M. P, Dunne JA Oxford: Oxford University Press 2006 27 86

[B32] Chaffron S Rehrauer H Pernthaler J von Mering C A global network of coexisting miobes from environmental and whole-genome sequence data Genome Res 2010 20 7 947 959 10.1101/gr.104521.109 20458099 PMC2892096

[B33] Guimera R Sales-Pardo M Amaral LA Classes of complex networks defined by role-to-role connectivity profiles Nat Phys 2007 3 1 63 69 10.1038/nphys489 18618010 PMC2447920

[B34] Brandes U Erlebach T Network analysis: methodological foundations 2005 Springer, Berlin

[B35] Bonacich P Power and Centrality - a Family of Measures Am J Sociol 1987 92 5 1170 1182 10.1086/228631

[B36] Watts DJ Strogatz SH Collective dynamics of 'small-world' networks Nature 1998 393 6684 440 442 10.1038/30918 9623998

[B37] Ravasz E Somera AL Mongru DA Oltvai ZN Barabasi AL Hierarchical organization of modularity in metabolic networks Science 2002 297 5586 1551 1555 10.1126/science.1073374 12202830

[B38] Costa LD Rodrigues FA Travieso G Boas PRV Characterization of complex networks: A survey of measurements Adv Phys 2007 56 1 167 242 10.1080/00018730601170527

[B39] West DB Introduction to Graph Theory 1996 Prentice Hall, Upper Saddle River, N.J.

[B40] Latora V Marchiori M Efficient behavior of small-world networks Phys Rev Lett 2001 87 19 198701 11690461 10.1103/PhysRevLett.87.198701

[B41] Wasserman S Faust K Social Network Analysis: Methods and applications 1994 Cambridge Univerisity Press, Cambridge

[B42] Krackhardt D Graph Theoretical Dimensions of Informal Organizations 1994 Lawrence Erlbaum and Associates, Hillsdale, NJ

[B43] Amaral LA Scala A Barthelemy M Stanley HE Classes of small-world networks Proc Natl Acad Sci USA 2000 97 21 11149 11152 11005838 10.1073/pnas.200327197 PMC17168

[B44] Girvan M Newman ME Community structure in social and biological networks Proc Natl Acad Sci USA 2002 99 12 7821 7826 10.1073/pnas.122653799 12060727 PMC122977

[B45] Newman MEJ Modularity and community structure in networks Proc Natl Acad Sci USA 2006 103 23 8577 8582 10.1073/pnas.0601602103 16723398 PMC1482622

[B46] Luo F Zhong J Yang Y Zhou J Application of random matrix theory to mioarray data for discovering functional gene modules Phys Rev E 2006 73 3 Pt 1 031924 10.1103/PhysRevE.73.031924 16605575

[B47] Ravasz E Barabasi AL Hierarchical organization in complex networks Phys Rev E 2003 67 2 026112 10.1103/PhysRevE.67.026112 12636753

[B48] Zhou J Wu L Deng Y Zhi X Jiang YH Tu Q Xie J Van Nostrand JD He Z Yang Y Reproducibility and quantitation of amplicon sequencing-based detection ISME J 2011 5 1303 1313 10.1038/ismej.2011.11 21346791 PMC3146266

[B49] He Z Xu M Deng Y Kang S Kellogg L Wu L van Nostrand JD Hobbie SE Reich P Zhou J Metagenomic analysis reveals a marked divergence in the structure of belowground miobial communities at elevated CO2 Ecol Lett 2010 13 5 564 575 10.1111/j.1461-0248.2010.01453.x 20337697

[B50] Nacke H Thurmer A Wollherr A Will C Hodac L Herold N Schoning I Schrumpf M Daniel R Pyrosequencing-Based Assessment of Bacterial Community Structure Along Different Management Types in German Forest and Grassland Soils PLoS ONE 201 6 2 e17000 21359220 10.1371/journal.pone.0017000 PMC3040199

[B51] Luo YQ Hui DF Zhang DQ Elevated CO2 stimulates net accumulations of carbon and nitrogen in land ecosystems: A meta-analysis Ecology 2006 87 1 53 63 10.1890/04-1724 16634296

[B52] Dethlefsen L Huse S Sogin ML Relman DA The Pervasive Effects of an Antibiotic on the Human Gut Miobiota, as Revealed by Deep 16 S rRNA Sequencing PLoS Biol 2008 6 11 2383 2400 10.1371/journal.pbio.0060280 PMC2586385 19018661

[B53] Alon U Biological networks: The tinkerer as an engineer Science 2003 301 5641 1866 1867 10.1126/science.1089072 14512615

[B54] Clauset A Moore C Newman ME Hierarchical structure and the prediction of missing links in networks Nature 2008 453 7191 98 101 10.1038/nature06830 18451861

[B55] Olesen JM Bascompte J Dupont YL Jordano P The modularity of pollination networks Proc Natl Acad Sci USA 2007 104 50 19891 19896 10.1073/pnas.0706375104 18056808 PMC2148393

[B56] Pons P Latapy M Computing communities in large networks using random walks Computer and Information Sciences - Iscis 2005, Proceedings 2005 3733 284 293 10.1007/11569596_31

[B57] Newman MEJ Finding community structure in networks using the eigenvectors of matrices Phys Rev E 2006 74 3 036104 10.1103/PhysRevE.74.036104 17025705

[B58] Guimera R Amaral LAN Cartography of complex networks: modules and universal roles J Stat Mech-Theory Exp 2005 2005 P02001 10.1088/1742-5468/2005/02/P02001 PMC2151742 18159217

[B59] Guimera R Amaral LAN Functional cartography of complex metabolic networks Nature 2005 433 7028 895 900 10.1038/nature03288 15729348 PMC2175124

[B60] Oldham MC Konopka G Iwamoto K Langfelder P Kato T Horvath S Geschwind DH Functional organization of the transiptome in human brain Nat Neurosci 2008 11 11 1271 1282 10.1038/nn.2207 18849986 PMC2756411

[B61] Horvath S Dong J Geometric interpretation of gene coexpression network analysis PLoS Comput Biol 2008 4 8 e1000117 10.1371/journal.pcbi.1000117 18704157 PMC2446438

[B62] Langfelder P Horvath S Eigengene networks for studying the relationships between co-expression modules BMC Syst Biol 2007 1 54 10.1186/1752-0509-1-54 18031580 PMC2267703

[B63] Butts CT Social Network Analysis with sna J Stat Softw 2008 24 6 1 51 18618019 10.18637/jss.v024.i01 PMC2447931

[B64] The igraph library [http://cneurocvs.rmki.kfki.hu/igraph/]

[B65] Langfelder P Horvath S WGCNA: an R package for weighted correlation network analysis BMC Bioinformatics 2008 9 559 10.1186/1471-2105-9-559 19114008 PMC2631488

[B66] Clauset A Newman ME Moore C Finding community structure in very large networks Phys Rev E 2004 70 6 Pt 2 066111 10.1103/PhysRevE.70.066111 15697438

[B67] Cline MS Smoot M Cerami E Kuchinsky A Landys N Workman C Christmas R Avila-Campilo I eech M Gross B Integration of biological networks and gene expression data using Cytoscape Nat Protoc 2007 2 10 2366 2382 10.1038/nprot.2007.324 17947979 PMC3685583

[B68] Maslov S Sneppen K Specificity and stability in topology of protein networks Science 2002 296 5569 910 913 10.1126/science.1065103 11988575

[B69] Bascompte J Jordano P Melian CJ Olesen JM The nested assembly of plant-animal mutualistic networks Proc Natl Acad Sci USA 2003 100 16 9383 9387 10.1073/pnas.1633576100 12881488 PMC170927

[B70] Bastolla U Fortuna MA Pascual-Garcia A Ferrera A Luque B Bascompte J The architecture of mutualistic networks minimizes competition and ineases biodiversity Nature 2009 458 7241 1018 1020 10.1038/nature07950 19396144

[B71] Bascompte J Jordano P Plant-animal mutualistic networks: The architecture of biodiversity Annu Rev Ecol Evol Syst 2007 38 567 593 10.1146/annurev.ecolsys.38.091206.095818

[B72] Arumugam M Raes J Pelletier E Le Paslier D Yamada T Mende DR Fernandes GR Tap J Bruls T Batto JM Enterotypes of the human gut miobiome Nature 2011 473 7346 174 180 10.1038/nature09944 21508958 PMC3728647

[B73] Fuhrman JA Miobial community structure and its functional implications Nature 2009 459 7244 193 199 10.1038/nature08058 19444205

[B74] Muegge BD Kuczynski J Knights D Clemente JC Gonzalez A Fontana L Henrissat B Knight R Gordon JI Diet Drives Convergence in Gut Miobiome Functions Aoss Mammalian Phylogeny and Within Humans Science 2011 332 6032 970 974 10.1126/science.1198719 21596990 PMC3303602

[B75] Bandyopadhyay JN Jalan S Universality in complex networks: Random matrix analysis Phys Rev E 2007 76 2 026109 10.1103/PhysRevE.76.026109 17930106

[B76] Plerou V Gopikrishnan P Rosenow B Amaral LAN Stanley HE Universal and nonuniversal properties of oss correlations in financial time series Phys Rev Lett 1999 83 7 1471 1474 10.1103/PhysRevLett.83.1471

[B77] Qin J Li R Raes J Arumugam M Burgdorf KS Manichanh C Nielsen T Pons N Levenez F Yamada T A human gut miobial gene catalogue established by metagenomic sequencing Nature 2010 464 7285 59 65 10.1038/nature08821 20203603 PMC3779803

[B78] Zhou X Kao MC Wong WH Transitive functional annotation by shortest-path analysis of gene expression data Proc Natl Acad Sci USA 2002 99 20 12783 12788 10.1073/pnas.192159399 12196633 PMC130537

[B79] Epstein SS Miobial awakenings Nature 2009 457 7233 1083 10.1038/4571083a 19242455

[B80] May RM Stability and complexity in model ecosystems, 1st Princeton landmarks in biology 2001 Oxford: Princeton University Press, Princeton, N.J

[B81] Kitano H Biological robustness Nat Rev Genet 2004 5 11 826 837 15520792 10.1038/nrg1471

[B82] Thebault E Fontaine C Stability of ecological communities and the architecture of mutualistic and trophic networks Science 2010 329 5993 853 856 10.1126/science.1188321 20705861

[B83] Wang F Zhou H Meng J Peng X Jiang L Sun P Zhang C Van Nostrand JD Deng Y He Z GeoChip-based analysis of metabolic diversity of miobial communities at the Juan de Fuca Ridge hydrothermal vent Proc Natl Acad Sci USA 2009 106 12 4840 4845 10.1073/pnas.0810418106 19273854 PMC2660763

[B84] Zhou J Kang S Schadt CW Garten CT Spatial scaling of functional gene diversity aoss various miobial taxa Proc Natl Acad Sci USA 2008 105 22 7768 7773 10.1073/pnas.0709016105 18509054 PMC2409408

[B85] Butte AJ Kohane IS Mutual information relevance networks: functional genomic clustering using pairwise entropy measurements Pac Symp Biocomput 2000 5 418 429 10902190 10.1142/9789814447331_0040

[B86] Carter DA Comprehensive strategies to study neuronal function in transgenic animal models Biol Psychiatry 2004 55 8 785 788 10.1016/j.biopsych.2003.07.005 15050858

[B87] Carlson MR Zhang B Fang Z Mischel PS Horvath S Nelson SF Gene connectivity, function, and sequence conservation: predictions from modular yeast co-expression networks BMC Genomics 2006 7 40 10.1186/1471-2164-7-40 16515682 PMC1413526

[B88] Wigner EP Random Matrices in Physics Siam Review 1967 9 1 1 10.1137/1009001

[B89] Mehta ML Random Matrices, 2nd edition Academic Press 1990

[B90] Zhong JX Geisel T Level fluctuations in quantum systems with multifractal eigenstates Phys Rev E 1999 59 4 4071 10.1103/PhysRevE.59.4071

[B91] Bohigas O Giannoni MJ Schmit C Spectral Properties of the Laplacian and Random Matrix Theories J Phys Lett-Paris 1984 45 21 1015 1022

[B92] Seba P Random matrix analysis of human EEG data Phys Rev Lett 2003 91 19 198104 14611622 10.1103/PhysRevLett.91.198104

[B93] Barabasi AL Scale-free networks: a decade and beyond Science 2009 325 5939 412 413 10.1126/science.1173299 19628854

[B94] Barabasi AL Albert R Emergence of scaling in random networks Science 1999 286 5439 509 512 10.1126/science.286.5439.509 10521342

[B95] Muller-Linow M Hilgetag CC Hutt MT Organization of excitable dynamics in hierarchical biological networks PLoS Comput Biol 2008 4 9 e1000190 10.1371/journal.pcbi.1000190 18818769 PMC2542420

[B96] Kirkpatrick S Gelatt CD Vecchi MP: Optimization by simulated annealing Science 1983 220 4598 671 680 10.1126/science.220.4598.671 17813860

[B97] Newman ME Fast algorithm for detecting community structure in networks Phys Rev E 2004 69 6 Pt 2 066133 10.1103/PhysRevE.69.066133 15244693

[B98] Olesen JM Bascompte J Dupont YL Jordano P The smallest of all worlds: pollination networks J Theor Biol 2006 240 2 270 276 10.1016/j.jtbi.2005.09.014 16274698

[B99] Alter O Brown PO Botstein D Singular value decomposition for genome-wide expression data processing and modeling Proc Natl Acad Sci USA 2000 97 18 10101 10106 10963673 10.1073/pnas.97.18.10101 PMC27718

[B100] Mantel N Detection of Disease Clustering and a Generalized Regression Approach Cancer Research 1967 27 2p 209 6018555

[B101] Dixon P VEGAN, a package of R functions for community ecology J Veg Sci 2003 14 6 927 930 10.1111/j.1654-1103.2003.tb02228.x

